# New treatments for hepatitis C virus (HCV): scope for preventing liver disease and HCV transmission in England

**DOI:** 10.1111/jvh.12529

**Published:** 2016-03-29

**Authors:** R. J. Harris, N. K. Martin, E. Rand, S. Mandal, D. Mutimer, P. Vickerman, M. E. Ramsay, D. De Angelis, M. Hickman, H.E. Harris

**Affiliations:** ^1^ Statistics, Modelling and Economics DepartmentPublic Health EnglandLondonUK; ^2^ Division of Global Public HealthUniversity of California San DiegoSan DiegoCAUSA; ^3^ School of Social and Community MedicineUniversity of BristolBristolUK; ^4^ University of PennsylvaniaPhiladelphiaPAUSA; ^5^ Immunisation, Hepatitis and Blood Safety DepartmentNational Infection ServicePublic Health EnglandLondonUK; ^6^ Liver UnitQueen Elizabeth HospitalBirminghamUK; ^7^ National Institute for HealthResearch Biomedical Research Unit and Centre for Liver ResearchUniversity of BirminghamBirminghamUK; ^8^ MRC Biostatistics UnitCambridge Institute of Public HealthCambridgeUK

**Keywords:** direct‐acting antivirals, hepatitis C virus, liver disease, people who inject drugs, prevention

## Abstract

New direct‐acting antivirals have the potential to transform the hepatitis C (HCV) treatment landscape, with rates of sustained viral response in excess of 90%. As these new agents are expensive, an important question is whether to focus on minimizing the consequences of severe liver disease, or reducing transmission via ‘treatment as prevention’. A back‐calculation model was used to estimate the impact of treatment of mild, moderate and compensated cirrhosis on incident cases of HCV‐related end‐stage liver disease/hepatocellular carcinoma (ESLD/HCC). In addition, a dynamic model was used to determine the impact on incidence and prevalence of chronic infection in people who inject drugs (PWID), the main risk group in England. Treating 3500 cirrhotics per year was predicted to reduce ESLD/HCC incidence from 1100 (95% CrI 970–1240) cases per year in 2015 to 630 (95% CrI 530–770) in 2020, around half that currently expected, although treating moderate‐stage disease will also be needed to sustain this reduction. Treating mild‐stage PWID was required to make a substantial impact on transmission: with 2500 treated per year, chronic prevalence/annual incidence in PWID was reduced from 34%/4.8% in 2015 to 11%/1.4% in 2030. There was little overlap between the two goals: treating mild stage had virtually no impact on ESLD/HCC within 15 years, but the long timescale of liver disease means relatively few PWID reach cirrhosis before cessation of injecting. Strategies focussing on treating advanced disease have the potential for dramatic reductions in severe morbidity, but virtually no preventative impact.

**Abbreviations**
CrI: credibility interval
DAA: direct‐acting antiviral
ELSD: end‐stage liver disease
HCC: hepatocellular carcinoma
HES: hospital episode statistics
IFN: interferon
ONS: Office of National Statistics
PWID: people who inject drugs
RIB: ribavirin

## Introduction

Liver disease is a growing public health problem in the UK, with a fivefold rise in liver‐related mortality in the under 65s since 1970 1. Chronic hepatitis C (HCV) infection contributes to a significant proportion of liver disease, with hospital admissions in England for HCV‐related end‐stage liver disease (ELSD) and hepatocellular carcinoma (HCC), representing prevalence of severe HCV‐related disease, rising from 574 in 1998 to 2652 in 2014; and reported deaths from these indications rising fourfold from 89 in 1996 to 362 in 2014 2. Unless HCV treatment is scaled up, it is likely that this trend will continue to beyond 2030, peaking at 1500 new cases of ESLD/HCC per year 3, placing a substantial burden on health care services and contributing to a marked reduction in lifespan.

The key risk group for HCV are people who inject drugs (PWID) with approximately 85% of prevalent infections due to injecting drug use in England 4. Therefore, any HCV intervention focusing on prevention of new infections requires targeting PWID; that is, those with an ongoing risk of transmission rather than those that have permanently ceased injecting. Prevalence of HCV antibodies in PWID is around 50% in England, but varies geographically from less than 20% to over 70% 5, 6. Primary prevention of HCV, such as needle and syringe programmes (NSPs) and opiate substitution therapy (OST), can prevent HCV transmission 7, but in isolation are unlikely to reduce HCV transmission to very low levels 8, 9. Modelling work has indicated that HCV treatment for PWID, combined with traditional harm reduction interventions, could be a cost‐effective means of HCV prevention, by substantially reducing transmission 9, 10, 11.

Historically, the number of people treated for HCV has been low. Of approximately 160 000 adults chronically infected in England 4, only around 5000 (3%) are treated per year 12, with numbers being treated declining for the first time since 2009 2. Recent estimates in several UK sites have shown very low treatment rates among PWID (5–20 per 1000 PWID annually) 6. These low treatment rates have been attributed to poorly tolerated interferon‐based therapies, with low efficacy in those with advanced disease and genotype 1 infection 13, the latter accounting for around 47% of infections in England 2. In the past year, however, the treatment landscape has been transformed by new direct‐acting antiviral (DAA) therapies, such as Sofosbuvir 14, Ledipasvir and various other new agents in combination undergoing approval. These new agents can be used in easy‐to‐administer, all‐oral regimens, which promise shorter treatment durations, improved side effect profiles and sustained viral response/cure rates exceeding 90% for genotype 1 infections 15, 16, and over 80% for genotypes 2 and 3 in clinical trials 17. Early results indicate that high SVR rates can also be achieved in the PWID population, due to good tolerance and short treatment durations 18. The list price of the new therapies, however, is considerable, with 12/24 week courses of Sofosbuvir at nearly £35 000/£70 000 14.

NHS England has recently announced a budget of £190 million for new treatments in 2015, primarily for treating those with cirrhosis 19. If budgets remain at this level in the near future, access will need to be carefully managed to ensure maximum clinical benefit and minimum harm. We therefore explored the potential impact of new treatments on incident cases of ESLD/HCC and on HCV incidence and chronic prevalence among PWID in the next 15 years, given different strategies for treatment scale‐ups in individuals with cirrhosis and pre‐cirrhotic disease stages.

## Materials and Methods

**Figure 1:**
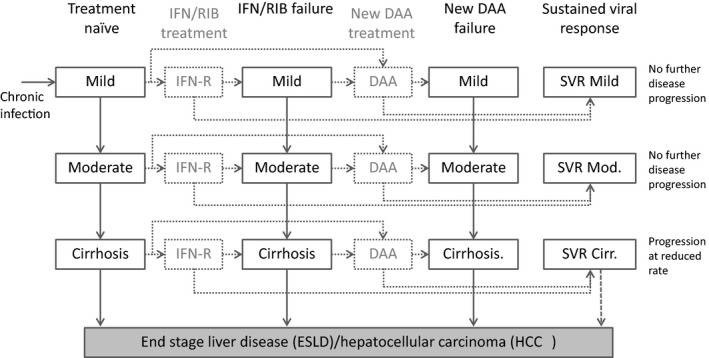
Multistate model representation of the natural history and treatment of HCV disease. For clarity, not all transitions are shown, as individuals who achieve SVR but are reinfected then return to their previous state prior to SVR. *IFN‐R*: pegylated interferon and ribavirin; *DAA*: direct‐acting antivirals.

We utilized two separate models for this analysis, based on previously published methods. The first was a back‐calculation model of HCV disease progression used to predict incident cases of ESLD/HCC in England, but does not include HCV transmission 3, 20. The second was a model of HCV transmission among PWID, which has been used to estimate the impact and cost‐effectiveness of HCV treatment scale‐up strategies 10. Both models incorporated a staged progression of HCV disease based on categories of modified HAI fibrosis stage 21 (Fig. 1), from infection through to mild chronic HCV (F0‐F2), moderate chronic HCV (F3‐F5), compensated cirrhosis (F6), ESLD (not including HCC), HCC and death (either subsequent to ESLD/HCC, or natural mortality). Individuals who are treated and achieve SVR in mild or moderate states were assumed to experience no further disease progression, while those that achieve SVR in the compensated cirrhosis state may still experience disease progression, but at a much reduced rate 22. Those failing treatment are not treated again with the same class of drugs. However, in the transmission model, reinfections may be treated again with the same class of drugs.

### Back‐calculation model to estimate end‐stage liver disease

Age‐specific data from Hospital episode statistics (HES) on ESLD and HCC, and Office of National Statistics (ONS) data on HCC mortality were used as disease endpoints. Information on the probabilities of progressing between disease states was taken from published literature and combined with the above data to derive estimates of the underlying HCV incidence and number of individuals in each disease state over time 3. Estimation is implemented in a Bayesian framework, and inference is expressed in terms of posterior distributions of the unknown quantities. These posterior distributions are obtained by simulation through Markov Chain Monte Carlo using WinBUGS 23. The median of the posterior distribution is taken to be the point estimate of the unknown quantity and 2.5th and 97.5th percentiles form a 95% credibility interval (CrI), the Bayesian equivalent of a confidence interval. The resulting posterior distributions of the disease‐state structure of the current infected population and progression probabilities are used in subsequent modelling for estimating future incident cases of HCV‐related ESLD/HCC (Appendix 1).

### Dynamic transmission model to estimate HCV chronic prevalence and incidence among PWID

We used a dynamic, deterministic, compartmental model of HCV transmission (Appendix 2) and disease progression (Fig. 1) among PWID to estimate the impact of HCV treatment among PWID (defined as those with an ongoing risk of transmission) in England. New injectors enter the population at risk of HCV infection through initiation of drug use and exit through permanent cessation of drug use or death. The key feature of this model is that HCV transmission is included in a dynamic way, such that the risk of HCV transmission and reinfection among PWID is related to the background level of transmission risk and prevalence of chronic infection among PWID. We make no assumption about any behaviour change after treatment, so that the incidence of primary infection equals that of reinfection. The model therefore quantifies the potential population benefits of reducing onward transmission via treatment, while accounting for risk of reinfection.

We assumed the risk of transmission or acquisition of HCV is independent of disease stage or duration of injecting. We also assumed that the HCV epidemic among PWID is at a stable steady‐state in 2015, based on the stable prevalence exhibited among PWID in contact with drugs services from 2003 to 2013 24, such that no changes in prevalence or incidence would occur without scale‐up of existing interventions. Upon infection, 25% spontaneously clear infection, with the remainder progressing to chronic infection, where individuals follow the natural history of disease progression and treatment as in Fig. 1.

The model was calibrated to estimates of the proportion of PWID with chronic HCV in England at an estimated 34% (95% CI 31–37%), based on 45% (95% CI 41–49) anti‐HCV prevalence in this group 4 and a 25% spontaneous clearance rate 25. This results in a median incidence of chronic infection/reinfection among PWID of 4.8 per 100 person‐years at baseline. We model a mean of 198 000 PWID in England (95% CrI 178 000–218 000) 4 and set the inflow rate of new PWID to match the outflow (due to death or cessation) such that the PWID population size is stable over time. The model was parameterized with UK estimates of drug‐related mortality 26, average duration of injection until permanent cessation 27 and rates of disease progression used in previous studies. To incorporate uncertainty in underlying parameters, each parameter was randomly sampled from its uncertainty range to produce 1000 parameter sets (Appendix 3). Each parameter set was used, and the transmission rate varied to calibrate the model to the sampled HCV prevalence among PWID in 2015. For each of these 1000 parameter sets, the model was run with varying levels of future treatments, and the median and 2.5th and 97.5th percentile projections are shown (95% Interval). The model was implemented in MATLAB.

### Treatment scenarios

Estimates of the impact of treatment were derived by assuming that a fixed number of individuals in different disease states receive treatment each year. We do not consider proportions currently diagnosed or increases in diagnosis that may be required to fulfil certain treatment scenarios, but place a 70% cap on the maximum proportion of infected individuals within a particular disease state that may be treated within any year.

Current standard treatment was assumed to be pegylated interferon (IFN) and ribavirin (RIB), which is assumed to continue to be used for patients that do not receive new DAAs. For the HCV disease burden model, age‐specific SVR rates for mild, moderate and cirrhosis stages were used from the Trent HCV cohort study 13, which recruits from all patients referred to participating centres in the Trent region and consists of around 70% infected via injecting drug use. In the HCV transmission model among PWID, estimates of IFN/RIB SVR rates among 500 PWID in the UK were used, which were consistent with published trials and observational studies among noninjectors 6. Rates are a weighted average over genotype 1 and non‐1, based on surveillance data 12.

DAA treatments were assumed to become available from 2015, with SVR rates of 90% for mild/moderate stages, and 80% for those with cirrhosis. These are somewhat lower than SVR rates observed in trials 16, 17 but real‐world rates are likely to be lower (as observed for IFN‐based regimens 13) given experiences so far in early access programmes 28. Current treatment for cirrhotics was assumed to be around 500 cirrhotics treated per year with IFN‐based treatment, which we compared with treating 3500 cirrhotics treated with new DAAs per year, matching the number expected to be treated under the new NHS budget 19. This would represent 40% of the estimated current number of individuals with compensated cirrhosis in England treated in the first year, compared to 6% per year currently 2. The population of untreated cirrhotics is therefore quickly exhausted, after which a maximum of 70% of the remaining untreated cirrhotic population were assumed to be treated per year. The 70% cap is used because it is likely that not all cirrhotics will be diagnosed and ready to start treatment and is based on the proportion anticipated to be eligible for treatment 2, 14, 29. The cap also reflects that upon developing cirrhosis treatment may not be initiated immediately, for example due to late diagnosis.

We then explored the impact of treating mild‐/moderate‐stage patients with new DAAs, assuming that roll‐out in these patients would start from 2016. The different scenarios are summarized in Box 1.

Box 1 **Scenarios for the scale‐up of new treatments in mild‐/moderate‐stage patients. all acenarios assume 3500 cirrhosis patients, up to a maximum of 70% of the remaining untreated cirrhotic population, are treated each year in England**

**Table:** 

**• DAAs for cirrhosis only:** Three thousand five hundred persons with cirrhosis, per year, up to 70% of the untreated cirrhotic population. About 50% of these are allocated as active PWID (up to a maximum of 70%). Maintain standard treatment at current rates for all other disease states: 2000 moderate and 2000 mild per year total, with 50% of these allocated as PWID (1000 moderate and 1000 mild).
**• DAAs for cirrhosis and moderate**: as in (1) plus 5000 persons with moderate‐stage infection receive new treatments per year from 2016. About 50% of these are allocated as active PWID in the transmission model (2500, up to a maximum of 70%).
**• DAAs all stages**: as in (2), plus 5000 persons with mild‐stage infection receive new treatments per year from 2016. About 50% of these are allocated as active PWID (2500, up to a maximum of 70%).

In the transmission model base‐case, half of the total number treated were allocated as active PWID. This assumption is intended to test the potential reductions in incidence that could be achieved. Similarly to the cap of 70% treated annually in cirrhotics, the maximum proportion of PWID treated per year in any stage was also set at 70%, as there are fewer moderate‐stage PWID. Awareness of infection in PWID is around 50% 2, so 70% assumes there will be improvements in diagnosis, retention, referral and treatment rates, and is probably the highest realistic level of treatment that could be reached in this population within the next 15 years.

A key assumption is that treatment with pegylated interferon and ribavirin continues for groups that do not receive DAAs. However, there may be reluctance among clinicians and patients to continue the use of IFN‐based therapy in the DAA era. We therefore examined the impact of the scenarios with scale‐up in new DAAs but no further use of IFN‐based regimens.

### Sensitivity analyses

We explored areas of uncertainty by varying key parameters in sensitivity analyses. There is currently little evidence on real‐world SVR rates for new DAAs by disease stage, so we compared scenarios with higher rates of 97.5% and 90% for all groups and lower rates of 90%, 80% and 70% in mild, moderate and cirrhosis groups, respectively. Post‐SVR progression rates are also uncertain; we tested the impact of assuming no post‐SVR progression in those with cirrhosis, and a scenario in which post‐SVR progression of cirrhotics was half that of non‐SVR (compared to 10 times lower from cirrhosis to ESLD and 4 times lower from cirrhosis to HCC at base‐case 22). Although the clinical benefit is unlikely to be as poor as merely halving disease progression, this represents a scenario where individuals continue to sustain liver damage, for instance due to alcohol abuse 30. For the PWID simulations, we additionally explore the impact of assuming different proportions of treatments allocated to PWID, such as 25% across all disease stages or 20% cirrhosis, 40% moderate, 60% mild as compared to 50% in our base‐case scenario.

## Results

### Reductions in incident cases of ESLD/HCC

**Figure 2:**
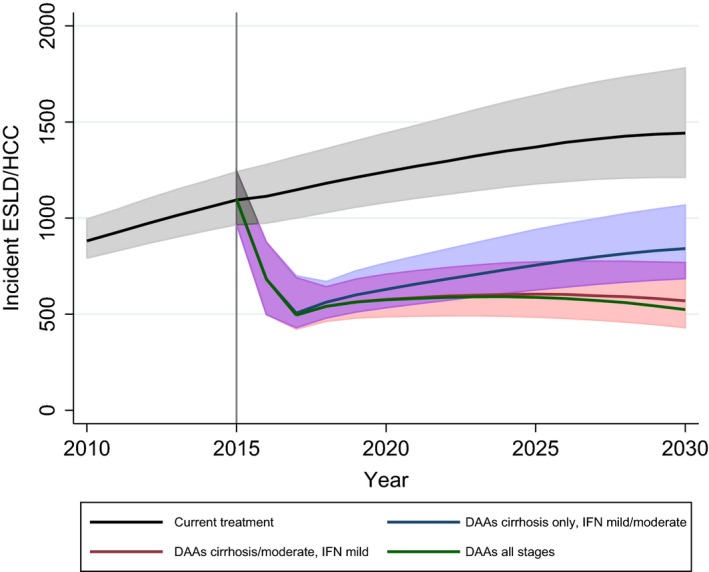
Predicted annual incident cases of end‐stage liver disease or hepatocellular carcinoma under the four main scenarios for DAA scale‐ups. 95% credible intervals are displayed where clarity permits*, with deeper shading for overlaps. *Credible interval for *DAAs all stages* not shown; uncertainty is of similar magnitude to other scenarios.

Treating 3500 cirrhotic patients with new DAAs each year (*DAAs for cirrhosis only*) from 2015 was predicted to reduce annual incidence of ESLD/HCC to 640 cases per year (95% CrI 530–770) in 2020 compared with 1240 under current levels of IFN‐based treatment (95% CrI 1080–1440) (Fig. 2). By 2017, the pool of untreated cirrhotics is less than 3000 individuals, and each year 70% of this group are treated subsequently; once treatment of cirrhotics reaches ‘saturation’, incident cases of ESLD/HCC begin to rise again, due to increasing numbers developing cirrhosis. In total, 6530 (95% CrI 5570–7980) cases of ESLD/HCC were predicted over 10 years (2016–2025) compared to 12 510 (95% CrI 10 980–14 620) under current levels of IFN‐based therapy, a reduction of 6000 (95% CrI 4910–7080) cases due to new treatments, or nearly 50%.

We tested whether the continued use of IFN‐based treatment made a difference to the predicted impact that new DAAs will have on ESLD/HCC. Without IFN‐based treatment for mild and moderate stages, there were predicted to be 21 (95% CrI 15–27) more incident cases of ESLD/HCC in 2020 (3.3% higher) and 59 (95% CrI 44–76) more in 2025 (7.8% higher). Further details are given in supplementary materials.

Treating 5000 moderates per year with new DAAs from 2016, in addition to cirrhotics (*DAAs for cirrhosis and moderate*), largely averts the predicted rebound in incident cases of ESLD/HCC, with incident cases not exceeding 600 per year and slowly beginning to decline from a maximum of 600 (95% CrI 480–770) in 2025 (Fig. 2). The total number of additional cases prevented over 10 years compared to treating cirrhotics only is 660 (95% CrI 500–850). The further scale‐up of DAAs for mild patients (*DAAs all stages*) had limited additional impact: virtually no difference was observed by 2020 compared to *DAAs for cirrhosis and moderate*, and even at 2025 the reduction in annual incident cases was only 16 cases (95% CrI 12–20). This reflects the long lead‐time before severe disease develops in those at mild stage.

### Reductions in incidence and prevalence of chronic HCV among PWID

At 2015, the model was calibrated such that chronic HCV prevalence among PWID was stable at a median of 34% (95% CrI 29–37%) with an annual incidence rate of 4.8% (95% CrI 3.6–8.1%). In this equilibrium state, the model predicts 79%, 16%, 5% in the mild, moderate, cirrhosis or later stages, respectively.

**Figure 3:**
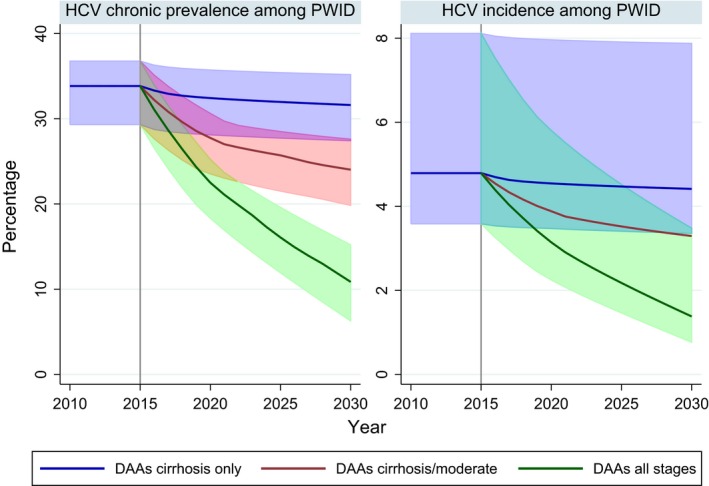
Estimated prevalence and incidence of chronic HCV among people who inject drugs in England with different treatment scenarios for DAA scale‐ups without interferon. Projections based on a starting scenario of 34% HCV chronic prevalence among PWID (95% CrI 30–37%) and 4.8 HCV incidence among PWID (95% CrI 3.6–8.1%). Lines show the median value, 95% uncertainty intervals are displayed where clarity permits*, with deeper shading for overlaps. *Uncertainty interval for *DAAs for cirrhosis/moderate* omitted from incidence plot; uncertainty is of similar magnitude to other scenarios.

Estimated prevalence and incidence of chronic infection among PWID are shown in Fig. 3. Treating cirrhosis alone with DAAs (*DAAs for cirrhosis only*) had negligible impact on predicted incidence and prevalence of chronic infection among PWID due to the rarity of advanced disease in this population. Only 7474 PWID (95% CrI 3834–11 782) were treated over the 15 years, and prevalence was reduced to 32% (95% CrI 27–35%) and incidence to 4.4% (95% CrI 3.6–7.9%) by 2030. With standard IFN/RBV treatment in mild and moderate stages (1000 per year in each) alongside treatment of cirrhotics with DAAs, prevalence was reduced to 25% (95% CrI 21–29%) and incidence to 3.5% (95% CrI 2.6–6.6%) by 2030. Treating 2500 moderate‐stage patients per year with DAAs (*DAAs for cirrhosis and moderate*) had some impact, with chronic prevalence estimated at 24% (95% CrI 20–28%) and incidence at 2.8%(95% CrI 2.1–5.7%) in 2030. However, by 2021, there are fewer PWID in the moderate stage than available treatments; in other words, as there are only 16% of PWID in the moderate stage, the pool of people to treat at this stage runs out. The additional scale‐up of DAAs to mild stage, however, with 2500 treated per year (*DAAs for all stages*) reduced chronic prevalence to 11% (95% CrI 6–15%) and incidence to 1.4% (95% CrI 0.7–3.5%) by 2030. In this scenario, nearly two‐thirds of the mild stage were treated, with a total of 68 179 PWID (95% CrI 61 236–75 210) treated over 15 years. It should be noted that the incidence rate of chronic infection is approximately 25% lower than the incidence rate of HCV exposure, due to spontaneous clearance 25.

### Sensitivity analyses

With better SVR rates, incident cases of ESLD/HCC are reduced by approximately the same degree in all scenarios, by around 60–80 cases per year (13% reduction *vs* base‐case in 2020) under 90% SVR rates for all disease stages and by 120–140 cases under 97.5% SVR rates (90% for mild/moderate and 80% for cirrhosis in the base‐case). With 90% SVR, marginal differences (<2%) are found for HCV prevalence among PWID in all scenarios. However, a 97.5% SVR results in 22% lower HCV prevalence among PWID in 2030 for the *DAA for all* scenarios compared to base‐case.

With worse SVR rates for moderate and cirrhosis stage (80% and 70%, compared to 90% and 80% in base‐case, respectively), incident ESLD/HCC was predicted to be around 80 cases higher (15% increase *vs* base‐case in 2020) for the *DAAs for cirrhosis only* scenario, with negligible difference (<1%) in HCV chronic prevalence among PWID in 2030 compared to base‐case. Cases of ESLD/HCC are around 130 higher for scenarios that treat pre‐cirrhotic stages, as the additional benefits of treating moderate stage are less. HCV chronic prevalence among PWID in 2030 is 5–10% higher compared to base‐case for pre‐cirrhotic scale‐up scenarios.

Changes to post‐SVR progression rates had a greater impact on incident cases of ESLD/HCC, although the variation in assumed values was fairly extreme. For *DAAs for cirrhosis only,* there were predicted to be up to 320 additional cases of ESLD/HCC in 2020 (51% higher than base‐case) if progression is only halved post‐SVR compared to the baseline rates, and up to 160 fewer cases if progression is halted entirely (25% lower than base‐case), although the difference increased over time. For scenarios that treat precirrhotic stages, changes in post‐SVR progression rates have less impact, as fewer patients progress to cirrhosis. No difference was seen in impact on HCV prevalence among PWID with variations in post‐SVR progression rates due to the very small number of PWID in later disease stages. Plots of predicted ESLD/HCC and prevalence/incidence of chronic HCV infection among PWID under the different sensitivity analyses are available in supplementary materials.

With fewer treatments allocated to PWID (25% compared to 50% at base‐case) there was less impact on HCV chronic prevalence among PWID, at 2030 a relative 13% higher in the *DAA for cirrhosis and moderates* scenario, and 74% higher in the *DAA for all stages* scenario (mean 19% chronic prevalence in 2030, 11% in base‐case). If 60%, 40%, 20% of mild, moderate and cirrhotic treatments, respectively, are allocated to PWID (50% at base‐case), then more impact is achieved in the *DAA for all stages* scenario, with HCV chronic prevalence 25% lower in 2025 (8% chronic prevalence in 2030, 11% in base‐case); marginal differences (<2%) are seen for other scenarios.

## Discussion

### Main findings

These analyses suggest that targeting new DAAs to people with cirrhosis will result in a rapid reduction in incident cases of ESLD/HCC within 2–3 years, to around half of that currently predicted in 2020, but with little impact on HCV incidence among PWID. Due to lower SVR rates in those with cirrhosis, continued post‐SVR progression (albeit at low rates) and rising numbers developing cirrhosis, treating those at moderate disease stage is also required to prevent a rebound in incident cases of ESLD/HCC. The total number of individuals either living with cirrhosis or having achieved SVR while cirrhotic was predicted to steadily increase, rising from 9330 (95% CrI 7090–12 020) in 2015 to 16 760 (95% CrI 13 730–21 000) in 2025, with an increasing number of moderate stage developing cirrhosis each year. Therefore, a rebound in ESLD/HCC (following ‘saturation’ of cirrhotic treatment) is to be expected unless individuals can be prevented from reaching cirrhosis in the first place. Re‐treatment may mitigate the increased risk of treatment failure in patients with cirrhosis to some extent, but there is still the risk of continued liver disease. The extent to which the risk of severe liver disease persists post‐SVR is not yet fully understood, although some studies suggest that fibrosis can be reversed 31. Sensitivity analyses indicated that even with SVR rates of virtually 100% (which could represent multiple rounds of treatment), without treating those at moderate‐stage ESLD/HCC will continue to rise beyond 2020 unless there is also no risk of post‐SVR progression. Clearly, there are potential risks to delaying treatment until patients develop cirrhosis.

New DAAs also offer the potential to reduce incidence and chronic prevalence in the PWID population by two‐thirds within 15 years. However, this requires treatment of those at mild stage, as the majority of PWID do not progress beyond mild stage during their injecting career. Treating PWID at moderate stage showed a 10% reduction in chronic prevalence, from 34% to 24%, but as a relatively small proportion of active PWID progress beyond mild stage, there are not enough moderate‐stage PWID to treat to make a substantial impact on transmission. This is in contrast to predicted incident cases of ESLD/HCC, which was only affected by treating moderate and cirrhotic stage disease within the time frame examined here.

### Strengths and limitations

By testing the impact of the same treatment scenarios within both a burden projection model and a dynamic transmission model, the impact on different aspects of harm reduction can be compared directly. This study provides the first population‐level assessment of the potential impact of new DAAs in England and the forthcoming NHS England treatment programme, quantified with total numbers treated in the infected population and in PWID. The proportion of treatments that are assumed to be ‘allocated’ to PWID is high, with the aim of assessing the potential impact rather than what might be feasible. The proportion of PWID treated will not affect the conclusion that the two reduction targets of transmission and severe disease have little overlap, but clearly if a lower proportion of those treated are active PWID, there will be a smaller reduction in transmission.

The disease burden model combines observed data on ESLD/HCC and HCC mortality from HES and the ONS, progression rates from the literature and estimates of overall population prevalence. The model should therefore provide an accurate picture of the current and future disease burden that England faces, and the potential impact of treatment. The main limitations are that there are no recent estimates of population prevalence; observed HES data may underreport HCV‐related ESLD/HCC to some extent; and the assumed model structure necessarily makes assumptions regarding relative risks of age‐specific progression and the distribution of age at infection.

The transmission model also has areas of uncertainty, primarily surrounding estimation of size of PWID population, natural history of injecting 32 and number of treatments allocated to PWID. For example, estimates of the PWID population range from less than 100 000 33 to nearly 200 000 34. Average prevalence of HCV in PWID in England is reasonably well established 2, 4 but subject to substantial local variation 5, 6, which greatly affects the impact of treatment as prevention 11; further, treatment rates in PWID vary substantially between settings 6. Length of injecting career is highly uncertain, with very limited data that can provide reliable estimates 27. We assume an average duration of 11 years, which leads to few PWID reaching cirrhosis before cessation. Disease progression in PWID has not been well studied; here, we have taken published estimates of disease progression used in previous modelling, which are consistent with those estimated from the burden model for the typical age range of this population. In any case, substantially higher rates in PWID would be required to alter the finding that cirrhosis is generally rare in this population.

We assume the risk of transmission or acquisition of HCV is independent of disease stage or duration of injecting. Several studies have noted that HCV prevalence rises quickly the first few years of injecting and then stabilizes 35, but heterogeneity in risk behaviour may also lead to rapid infection in high‐risk individuals, with infections occurring more slowly in lower‐risk groups subsequently. One UK‐based modelling study 36 indicated strong population heterogeneity in risk behaviour, but also an elevated risk of HCV acquisition within the first year of injecting. By contrast, a recent study from Australia 32 showed that while the risk of injecting cessation increases over time, marginal differences were seen in risk behaviours among those who remain injecting. If injecting risk is heightened within the first year of injecting and HCV treatment reaches PWID later in their career, this would reduce the population‐level impact projected here. Nevertheless, our findings support previous modelling work indicating that the existing level of HCV treatment in the UK is unlikely to result in measurable impact among PWID, but that scale‐up could dramatically reduce chronic prevalence among PWID 6, 11, 37.

Key uncertainties include the actual SVR rates of new treatments in practice, which are likely to be worse than those observed in clinical trials, and the lack of long‐term outcome data in those that achieve SVR where cirrhosis has already developed. Crucially, it is also uncertain to what extent SVR rates are reduced in patients with cirrhosis compared to pre‐cirrhotic disease stages. Finally, it is not known to what extent treating DAA‐failures with other regimens will be viable, with issues such as cross‐class drug resistance still being investigated. Our base‐case assumption was for no re‐treatment, but we sought to consider alternatives by testing assumptions of higher overall SVR rates, and other uncertainties listed above, in sensitivity analyses.

### Implications

These analyses show that the major concerns relating to HCV, rising severe liver disease and ongoing transmission, can be addressed with the advent of new DAAs. Treating those with cirrhosis fulfils the short‐term goal of reducing ELSD/HCC, although treatment of moderate stage is needed to prevent a subsequent rebound. Treatment of mild‐stage, active PWID is required to substantially reduce HCV transmission, which must be addressed if the long‐term goal of elimination of HCV as a significant public health concern is to be achieved.

Progress towards reducing ESLD/HCC should be immediately observable via HES data and liver‐related mortality, although reductions in transmission will be harder to detect. Changes in incidence cannot easily be observed directly, and falling chronic prevalence will occur slowly and also be difficult to monitor accurately, with current monitoring via anonymous surveys testing for antibodies 38. A trial of ‘treatment as prevention’ is therefore desirable to demonstrate an intervention effect and to establish what data collection systems are required to monitor progress. Collecting national treatment uptake and outcomes data are crucial for monitoring progress and predicting likely impact. Currently no national data collection for HCV treatment is undertaken and numbers are only estimated indirectly 2, with real‐world SVR rates only available from localized cohort studies. Fortunately, monitoring is set to improve with the roll‐out of new therapies.

With a budget of £190 million for new DAAs announced in June 2015 and current focus on treating those with cirrhosis 19, only the short‐term goal of reducing ESLD/HCC over the next 5 years can be achieved. Clearing the current pool of those diagnosed with cirrhosis could be achieved within 2–3 years at the treatment levels here. With recent NICE approval for use in all stages of genotype 1 infection, treatment of pre‐cirrhotic stages should follow shortly. Reductions in HCV transmission, however, will require a change in strategy to treat substantial numbers of active PWID, most of whom will have relatively mild disease.

The key issue for any managed, targeted treatment strategy is whether individuals in the appropriate groups can be identified and treated in a timely way. One potential strategy would be to monitor patients using fibrosis scans and only initiate treatment when a defined high‐risk fibrosis stage is reached 39. However, limitations of current technology mean that there is a chance that some high‐risk individuals may be missed. In addition, marginalized groups may become disengaged from long‐term monitoring (or ‘watchful waiting’) programmes and miss out on future treatment opportunities, therefore widening health inequalities. Likewise, it is uncertain whether there is any place for ongoing use of standard treatments as a first‐line approach from practical, ethical or patient/clinician acceptability standpoints.

The scenarios here also require a certain quota of people to be treated each year. While it is likely that the majority of those with cirrhosis will be diagnosed, treating 70% of the remaining pool each year may be difficult to achieve in practice. Late diagnosis of cirrhosis will result in continued high levels of severe liver disease. Treating 5000 mild and 5000 moderate per year would be a relatively small proportion of the current infected population, but as the pool of diagnosed individuals diminishes, sustaining high numbers in treatment may be difficult unless testing, diagnosis and patient engagement improve. Diagnosing and treating sufficient numbers of PWID may be difficult, although recent advancements in case‐finding through dried blood spot testing and voluntary opt‐out in prison are likely to increase diagnosis rates in this population 40, 41. New methods of treatment delivery, such as community settings, may need to be expanded to achieve the treatment levels among PWID that are modelled here.

## Conclusions

With the arrival of new DAA treatments, the outlook is extremely good for HCV‐infected patients, with the risk of severe HCV‐related liver disease being markedly reduced. However, SVR rates in cirrhotic patients may be lower than pre‐cirrhotic patients, treatment of cirrhosis may not prevent further disease progression in all individuals, and treatment of cirrhotic patients will have little benefit on preventing new HCV infections. Therefore, focussing solely on cirrhotics is not a tenable long‐term strategy if continued reductions in incident cases of ESLD/HCC and reductions in transmission are to be achieved. NHS England is currently rolling out new DAAs to those with cirrhosis, but treatment of other groups will need to follow quickly. Providing equitable access to expensive treatments will require a difficult balance between increasing the budget for treatment and negotiating lower prices.

## Supporting information


**Data S1.** Materials.

## Appendix 1

### Back‐calculation approach

Back‐calculation incorporates knowledge of progression probabilities taken from the literature (*prior* distributions) which are combined with the observed data to produce *posterior* distributions for the quantities of interest. The approach is based on the observation that in a disease with long incubation period, there is a long delay between infection and the development of the disease endpoint 42. Let *t*_0_ < *t*_1_… < *t*_*N*_ partition time between *t*_0_ and *t*_*N*_ into intervals of equal length. Then, in discrete time, this idea can be analytically expressed through the relation

(1) $$\mu_{i}=\sum_{j=0}^{i}h_{j}f_{j-i}$$

linking the three components of the back‐calculation: µ_*i*_, the expected number of occurrences of the endpoint of interest in the time interval [*t*_*i*_, *t*_*i*+1_]; *h*_*j*_ the expected number of new infections during [*t*_*j*_, *t*_*j*+1_]; and *f*_*j*−*i*_, the probability that infections in the interval [*t*_*j*_, *t*_*j*+1_] experience the endpoint in [*t*_*i*_, *t*_*i*+1_] for *i* = 1 … *N* − 1. So, given data on the endpoint over time and knowledge of the distribution of the time between infection and endpoint, it is possible to reconstruct the temporal pattern of the new infections. Estimation typically proceeds by assuming that the underlying, unobserved, infection process is Poisson; that is the number of new infections in any interval [*t*_*j*_, *t*_*j*+1_] has a Poisson distribution with mean *h*_*j*_. From this, through (1), the number of new cases of the endpoint in the interval [*t*_*i*_, *t*_*i*+1_] is Poisson distributed with mean µ_*i*_. The distributional assumption allows the derivation of the likelihood of the observed data over the period between *t*_0_ and *t*_*N*_ as a product of Poisson variables whose means are functions of the unknown *h*_*j*_. By suitably expressing the *h*_*j*_ in terms of parameters θ, that is *h*_*j*_ = *h*_*j*_(θ), the maximization of the likelihood provides estimates of the parameters and of the number of new infections over time.

A Bayesian approach to this estimation involves the specification of a prior distribution on θ. This prior can then be combined with the likelihood to produce a posterior distribution reflecting both a priori knowledge and the information from the observed data. The combination of these two information components is carried out using Bayes' theorem, according to which

(2) p(θ|data)∝p(θ)×p(data|θ)

where *p*(θ) is the prior information on θ, *p*(data|θ) is the likelihood of the data and *p*(θ|data) represents the posterior distribution of θ after observing the data. Summaries of the posterior distributions, specifically medians, are taken as point estimates, and the 2.5th and 97.5th percentiles forming the credible interval (CrI), the Bayesian equivalent of a 95% confidence interval.

In this article, endpoint data are based on hospital admissions for HCV‐related end‐stage liver disease (ESLD) and hepatocellular carcinoma (HCC), which are obtained from Hospital Episode Statistics (HES) data; and HCV‐related HCC deaths from the ONS. Given that ESLD and HCC are serious conditions requiring urgent medical attention, the HES data are taken to be a measure of population‐level prevalence of these conditions. HCV infection status for HCC deaths is taken to be under‐reported and corrected for using the method described by Sweeting *et al*. 20. The observed data are in the form of aggregate counts of number occupying these disease states (or the incident occurrence, for deaths) each year, according to 10‐year age groups. Summaries of observed hospital admissions for end‐stage liver disease and hepatocellular carcinoma, and mortality, are available from previous Public Health England reports (2).

The resulting output from the model therefore includes posterior distributions of the progression rates and the number of individuals within each disease state over time. This output thus forms the basis of future projections, with the numbers in each disease state at time *t* + 1 derived from the infected population structure at the current time, *t* and rates of progression to subsequent stages. Age‐specific prior and posterior distributions for the progression probabilities are shown below.

**Table:**

	Annual probability (95% CrI)	
	Prior	Posterior
Acute to chronic infection		
	0.7376 (0.6984, 0.7730)	0.7376 (0.6984, 0.7730)
Chronic HCV to moderate chronic HCV		
0–9	0.0204 (0.0117, 0.0331)	0.0161 (0.0085, 0.0278)
1–10		0.0197 (0.0116, 0.0306)
20–29		0.0158 (0.0092, 0.0238)
30–39	0.0089 (0.0029, 0.0218)	0.0115 (0.0045, 0.0204)
40–49	0.0156 (0.0056, 0.0338)	0.0425 (0.0249, 0.0637)
50–59	0.0534 (0.0345, 0.0782)	0.0523 (0.0337, 0.0776)
60–69		0.0593 (0.0379, 0.0843)
70–79		0.0667 (0.0459, 0.0921)
80+		0.0546 (0.0354, 0.0787)
Moderate chronic HCV to cirrhosis		
0–9	0.0075 (0.0010, 0.0258)	0.0001 (0.0000, 0.0004)
1–10		0.0023 (0.0013, 0.0047)
20–29		0.0074 (0.0048, 0.0134)
30–39	0.0036 (0.0001, 0.0219)	0.0168 (0.0109, 0.0267)
40–49	0.0065 (0.0004, 0.0271)	0.0261 (0.0189, 0.0392)
50–59	0.0282 (0.0062, 0.0786)	0.0142 (0.0076, 0.0264)
60–69		0.0293 (0.0204, 0.0398)
70–79		0.0563 (0.0360, 0.0831)
80+		0.1355 (0.0919, 0.1994)
Cirrhosis to hepatocellular carcinoma		
0–29	0.0079 (0.0040, 0.0159)	0.0068 (0.0043, 0.0093)
30–39	0.0130 (0.0075, 0.0219)	0.0113 (0.0080, 0.0148)
40–49	0.0212 (0.0142, 0.0311)	0.0186 (0.0145, 0.0233)
50–59	0.0347 (0.0249, 0.0475)	0.0307 (0.0247, 0.0385)
60–69	0.0565 (0.0381, 0.0792)	0.0513 (0.0402, 0.0671)
70+	0.0913 (0.0561, 0.1469)	0.0844 (0.0639, 0.1211)
Cirrhosis to decompensated cirrhosis (end‐stage liver disease)		
0–29	0.0651 (0.0139, 0.2610)	0.1490 (0.0804, 0.2230)
30–39	0.0641 (0.0219, 0.1750)	0.1286 (0.0801, 0.1793)
40–49	0.0648 (0.0324, 0.1186)	0.1123 (0.0800, 0.1465)
50–59	0.0649 (0.0403, 0.0951)	0.0974 (0.0763, 0.1254)
60–69	0.0635 (0.0336, 0.1186)	0.0851 (0.0681, 0.1111)
70+	0.0630 (0.0229, 0.1675)	0.0748 (0.0558, 0.1010)
Decompensated cirrhosis to hepatocellular carcinoma		
0–29	0.0155 (0.0074, 0.0328)	0.0114 (0.0034, 0.0222)
30–39	0.0252 (0.0137, 0.0440)	0.0186 (0.0065, 0.0346)
40–49	0.0410 (0.0248, 0.0644)	0.0305 (0.0120, 0.0548)
50–59	0.0665 (0.0416, 0.1026)	0.0502 (0.0228, 0.0884)
60–69	0.1091 (0.0646, 0.1751)	0.0835 (0.0419, 0.1427)
70+	0.1762 (0.0945, 0.3251)	0.1371 (0.0730, 0.2300)
Decompensated cirrhosis to liver‐related mortality (not hepatocellular carcinoma)		
	0.1857 (0.1289, 0.2556)	0.2930 (0.2288, 0.3576)
Hepatocellular carcinoma to death specific to hepatocellular carcinoma		
	0.6032 (0.5323, 0.6774)	0.6026 (0.5510, 0.6541)

## Appendix 3

### Parameters for HCV transmission among PWID

**Table:**

	Mean value	Distribution	Reference
Disease state transition probabilities per year (probabilities converted to instantaneous rates)			
Mild HCV to moderate HCV	0.025	Beta (α = 38.0859, β = 1485.3516)	43
Moderate HCV to compensated cirrhosis	0.037	Beta (α = 26.905, β = 700.2582)	43
Compensated cirrhosis to ESLD	0.039	Beta (α = 14.6168, β = 360.1732)	43
Compensated cirrhosis to ESLD SVR, relative risk of non‐SVR	7%	Lognormal (95% CI 0.03, 0.20)	44, 45
Compensated cirrhosis/ESLD to HCC	0.014	Beta (α = 1.9326, β = 136.1074)	43
Compensated cirrhosis to HCC SVR, relative risk of non‐SVR	23%	Lognormal (95%CI 0.16, 0.35)	45
ESLD to death	0.13	Beta (α = 147.03, β = 983.97)	43
HCC to death	0.43	Beta (α = 117.1033, β = 155.23)	43
Epidemiological parameters			
Number of PWID in England	198 000	Normal (95%CI 178 000–218 000)	4
HCV chronic prevalence among PWID	34%	Normal (95%CI 31–37%)	4
Average injecting duration until permanent cessation (years)	11	Uniform (6,16)	27
Average PWID excess death rate per year	0.01	Poisson	26
Proportion genotypes 1 and 4	50%		46
Proportion genotypes 2 and 3	50%		46
PWID SVR genotypes 1 and 4 with IFN/RIB	45%	Uniform (33–57%)	6
PWID SVR genotypes 2 and 3 with IFN/RIB	61%	Uniform (47–76%)	6
