# Supplementary material for: New treatments for hepatitis C virus (HCV): scope for preventing liver disease and HCV transmission in England
Source: J Viral Hepat. 2016 Mar 29;23(8):631–43. doi: 10.1111/jvh.12529 (PMC4982023; doi:10.1111/jvh.12529)
Supplement: Supplementary file 1 — Data S1. Materials. [file JVH-23-631-s001.docx]

## Supplementary materials

## 1. Sensitivity analyses of HCV burden

### SVR rates

Different SVR rates for new DAAs (higher and lower) vs. baseline in the four treatment scenarios and impact on predicted incident cases of ESLD/HCC. Baseline SVR is 90% for mild and moderate disease stage, 80% for cirrhosis (cirr). Varying the rate makes no difference to the scenario for no DAAs, but is shown for completeness.

### Post-SVR progression

Post-SVR progression rates are around 10 times lower for developing ESLD and 4 times lower for HCC. Shown below are results for progression only being halved, and no further progression, compared to the baseline for the four scenarios and impact on predicted incident cases of ESLD/HCC.

### Standard treatment ceases

The baseline scenario assumes that pegylated interferon and ribavirin will continue to be used for disease stages that do not receive new DAAs. These analyses examine the impact on the four treatment scenarios if those not receiving new DAAs receive no treatment. There is no difference for the scenario where all disease stages receive DAAs, but this is shown for completeness.

## 2. Sensitivity analyses of HCV transmission

The base case was for 90%/90%/80% SVR rates in mild, moderate and cirrhosis stages, with 50% of treatments in the infected population allocated to people who inject drugs (PWID). Sensitivity analyses explored higher and lower SVR rates, no post-SVR disease progression in cirrhotics vs. a reduction in progression of just 50% post-SVR, and different proportions of the treated population allocated as active PWID. The plot below shows the resulting impact of different assumptions on prevalence and incidence of chronic HCV in PWID in 2030 according to the different scenarios for treatment.


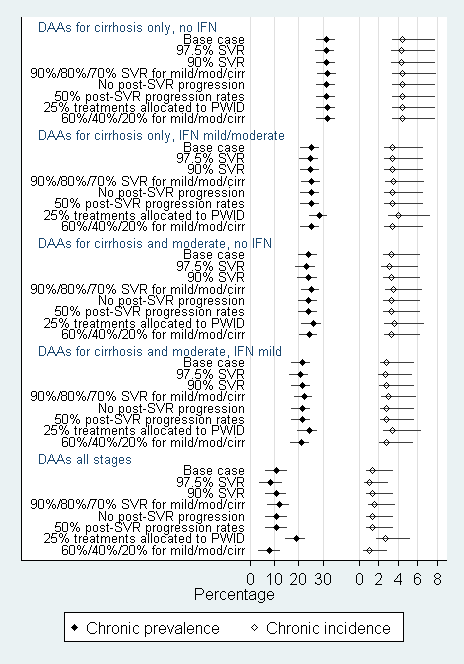


*mod* = moderate, *cirr* = cirrhosis
